# Modulation of redox reactivity of resazurin through host-guest complexation with Cucurbit[*n*]uril (*n* = 7, 8)

**DOI:** 10.3389/fchem.2023.1295715

**Published:** 2023-12-14

**Authors:** Jia Liu, Su-Hang He, Hugues Lambert, Tung-Chun Lee

**Affiliations:** ^1^ Department of Chemistry, University College London (UCL), London, United Kingdom; ^2^ Institute for Materials Discovery, University College London (UCL), London, United Kingdom; ^3^ Xiamen Institute of Rare-earth Materials, Haixi Institutes, Chinese Academy of Sciences, Xiamen, China; ^4^ Center of Single-Molecule Sciences, College of Electronic Information and Optical Engineering, Nankai University, Tianjin, China

**Keywords:** supramolecular electrochemistry, host-guest complexes, reactivity modulation, resazurin, cucurbituril

## Abstract

Resazurin (Alamar Blue, RZ) is a widely utilized fluorescent probe for biological sensing, whose fluorescent intensity can be modulated by changing its redox states; thereby, electrochemical reactivity of RZ is of significance when designing a sensing assay. Herein, we report novel two-way electrochemical reactivity modulation of RZ using host-guest complexation with rigid molecular containers cucurbit[*n*]uril (CB*n*, *n* = 7, 8). The complexation between CB*n* and RZ is confirmed by ^1^H NMR measurements and supported by computational simulation, and the binding constants are determined via UV-vis titration. Notably, the voltametric data highlights that the redox reactivity of RZ can be activated or deactivated upon encapsulation by CB8 or CB7, respectively. This two-way reactivity modulation is hypothesized to be mediated by the difference in cavity volume that favors or hinders the approach of water molecules to the encapsulated reaction center during the reduction process. Despite the similar cavity size to CB, molecular containers such as cyclodextrins (CDs) exhibit considerably weaker modulation effects. Our approach can potentially be applied to other redox processes that involve proton transfer, and open new possibilities in supramolecular electrochemistry.

## 1 Introduction

Resazurin (7-hydroxy-3H-phenoxazin-one-10-oxide, RZ), also known as Alamar Blue, is a redox-sensitive, non-toxic, and water-soluble dye that has found widespread use as a tracer for analyzing diverse biochemical and metabolic processes in biological system (O'Brien et al., 2000; Emter and Natsch, 2015; Präbst et al., 2017; Mishra et al., 2019). The versatility of RZ as a redox dye stems from its two-step reduction process. Initially, the purple and slightly fluorescent RZ undergoes an irreversible reduction, transforming into pink and highly fluorescent resorufin (RS). This step is then followed by a reversible reduction, converting RS into a colorless and non-fluorescent compound known as dihydro-resorufin (DHRS) (Ibáñez et al., 2020). The modulation of these reduction processes is beneficial for exploiting RZ as a sensing indicator; however, it remains largely unexplored in existing literature, highlighting its untapped potential and practical significance.

Cucurbitu[*n*]rils (CB*n*, *n* = 5–8) are a family of macrocyclic hosts synthesized through the condensation of affordable starting materials (Barrow et al., 2015; Lin et al., 2020). Their versatile properties offer a wide range of potential applications, including reactivity modulation (Lee et al., 2013; Moorthy et al., 2022; Moorthy et al., 2023), drug delivery (Walker et al., 2011), environmental monitoring (Chio et al., 2022), explosive detection (Chio et al., 2019) and biosensing (Chio et al., 2020; Chio et al., 2021; Davison et al., 2023). Notably, CB7 and CB8, are both water-soluble (with a solubility of >20 mM for CB7 and <0.1 mM for CB8, depending on the preparation method) and highly biocompatible, demonstrate promising applicability in aqueous-based scenarios, such as biological systems (Uhlenheuer et al., 2010) and electrochemical investigations (Boulas et al., 1998; Jeon et al., 2002; Gadde et al., 2009; Luo et al., 2019). The electrochemical behaviors of CB7- and CB8-encapsulated redox-active molecules, *e.g.*, dimethyl viologen (MV^2+^), have been reported by Kim et al. (Jeon et al., 2002; Kim et al., 2002) and Kaifer et al. (Ong et al., 2002; Ling et al., 2007; Gadde and Kaifer, 2011). In particular, encapsulation of MV^2+^ within CB7 was observed to deactivate its reduction reaction due to the preferential stabilization of the dicationic charge by the electron-rich portals of CB7. In contrast, the reduction of MV^2+^ can be significantly activated upon CB8-encapsulation by forming stable 1:2 complexes of CB8•2 MV^+•^. However, this activation driven by dimerization relies on the formation of highly polarizable aromatic radical cations (*e.g.*, MV^+•^) (Cai et al., 2021), which are relatively rare. Thus, precedent examples are limited to only a few viologen-based compounds.

**FIGURE 1:**
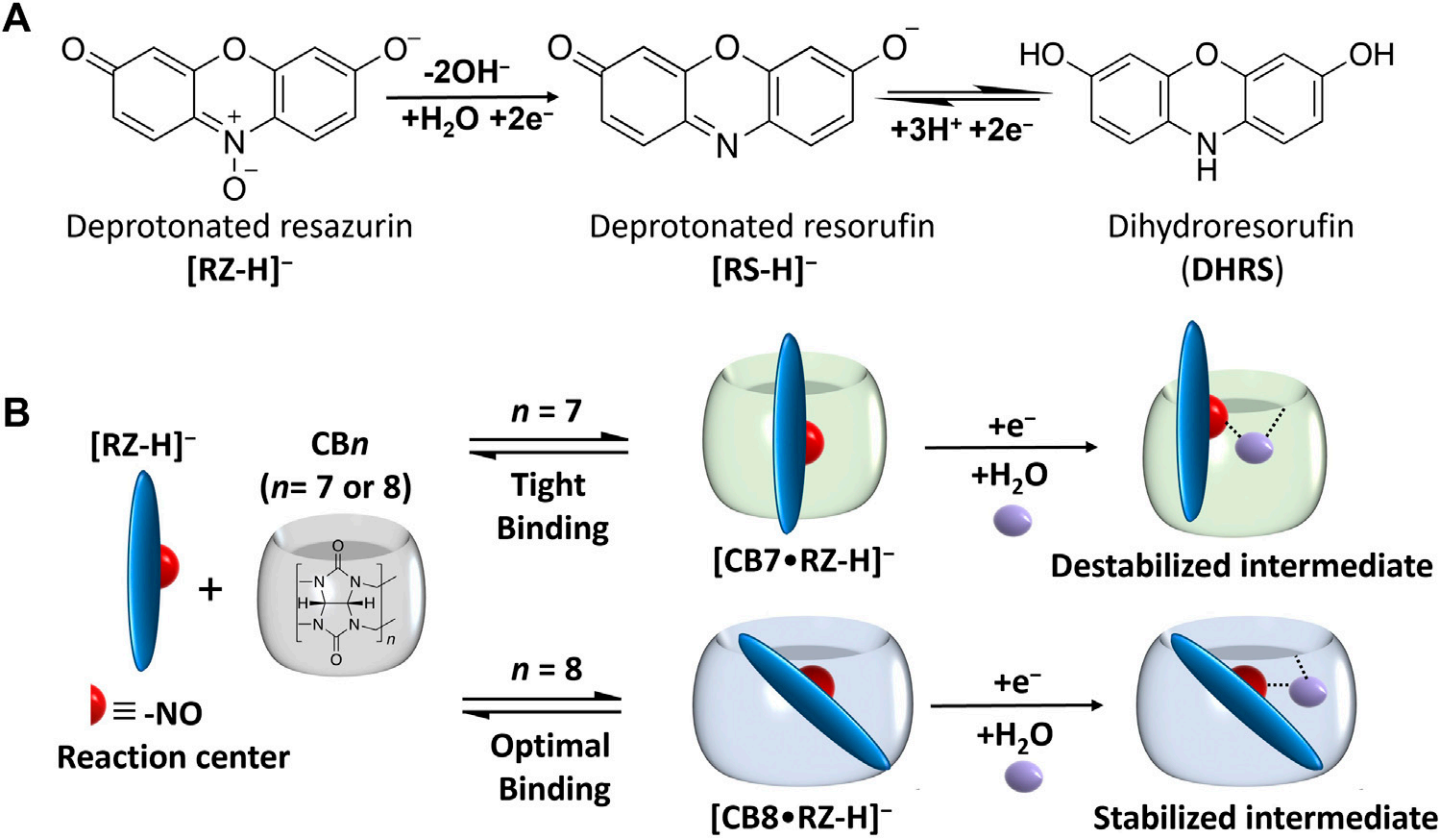
**(A)** Reduction process of deprotonated resazurin ([RZ-H]^–^) and corresponding chemical structures at pH 7.5 **(B)** Host-guest complexation process of [RZ-H]^–^ with CB7 and CB8 and schematic illustration of corresponding intermediates formed by the first one-electron reduction process.

Herein, we report the host-guest complexation of resazurin with CB7 and CB8 for the first time. UV-vis spectroscopy and NMR spectroscopy were used to determine the binding affinities of the inclusion complexes, while computational modelling based on density functional theory (DFT) was employed to investigate the binding modes. Notably, we discovered that the electrochemical reduction of resazurin can be either activated or deactivated through complexation with different cucurbituril homologues. Specifically, CB7 was found to deactivate the reduction process (Δ*E*_CV_ = −54 mV), whereas CB8 activated it (Δ*E*_CV_ = +37 mV), as confirmed by cyclic voltammetry (CV) and square wave voltammetry (SWV) measurements. DFT modelling provided insights into the underlying mechanism, suggesting that the variation in cavity volume facilitates or hinders the approach of water molecules to the encapsulated reaction center, i.e., the -NO group (Figure 1). Our supramolecular approach holds promising prospects for a broader range of redox-active host-guest complexes, where proton transfer is coupled with electron transfer during electrochemical reactions, and thus open new possibilities in supramolecular electrochemistry.

## 2 Materials and experimental methods

### 2.1 Materials

Resazurin, boric acid and phosphoric acid were purchased from Aladdin. Acetic acid and sodium hydroxide were bought from SCR. Resorufin was ordered from TCI. β-cyclodextrin (βCD), γ-cyclodextrin (γCD), deuterium oxide, deuterium chloride (20% in D_2_O) and 1-adamantylamine (AdNH_2_) were bought from Adamas. Cucurbit[7]uril (CB7) and cucurbit[8]uril (CB8) were synthesized and purified following the procedures reported before (Isaacs, 2009). All chemicals were of analytical grade and used without further treatment.

### 2.2 Electrochemical measurements

All electrochemical experiments were performed on Gamry Interface 1010E workstation. A glassy carbon working electrode (d = 3 mm), platinum plate counter electrode (6.5 mm × 6.5 mm) and leakless Ag/AgCl reference electrode (d = 5 mm) were inserted into a 50-mL single-compartment electrochemical cell. Prior to each electrochemical measurement, all samples were purged with nitrogen gas for more than 10 min to remove dissolved oxygen in solution, and the surface of glassy carbon working electrode was polished with a 0.05 μm alumina slurry on a felt polishing cloth. To eliminate the influences of pH on the electrochemical behaviors of RZ, which is pH-sensitive, pH values of all samples were adjusted again after introducing host molecules into the sample solution.

CV measurements were conducted by sweeping potential from 300 mV to −400 mV at a scan rate of 10 mV/s with a step size of 1 mV for 5 cycles. The SWV measurements were performed with initial potential of 300 mV, final potential of −400 mV, step size of 1 mV, pulse size of 25 mV and frequency of 5 Hz. Unless stated otherwise, all measurements were done with these parameters.

All samples for electrochemical measurements were prepared at concentration of 1 mM in Britton-Robinson (B-R) buffer at pH of 7. It should be noted that the [CB8•RZ-H]^−^ complex formation might lead to a slight reduction in the solubility of both the host and guest molecules in the solution, resulting in the formation of a minor amount of precipitate. However, the precipitate was removed through filtration prior to the measurements. B-R buffer solution was prepared by adjusting the pH of a mixture of boric acid (0.04 M), phosphoric acid (0.04 M) and acetic acid (0.04 M) by NaOH (0.2 M). The pH of solution was checked by Mettler Toledo F20 pH meter.

### 2.3 ^1^H NMR measurements

^1^H NMR spectra were collected using a Bruker Avance III 500 MHz spectrometer. Samples for ^1^H NMR measurements were prepared at 1 mM in D_2_O unless specified otherwise.

### 2.4 UV-vis titrations

UV-vis titrations for the determination of binding constants were conducted using an Agilent Cary 60 spectrophotometer. The guest molecule was maintained at a concentration of 4 μM throughout the titration, while the concentration of the host molecule was gradually increased. Water was used as the solvent.

### 2.5 Optimization of molecular models

Initial optimization of molecular models was done by MMFF94 in Chem3D, and the full optimization was performed at wB97XD/6-31G* and CPCM/wB97XD/6-31G* level of theory using Gaussian 09. Solvent effects were approximated by using CPCM implicit water model. Moreover, the van der Waals interactions, which are regarded as the major contributor for the stability of the host-guest complexes, was evaluated by employing the dispersion-corrected DFT functional wB97XD. Energy change in redox reactions and complexation-decomplexation processes were calculated by using the energy of optimized models. All DFT optimization was performed on the UCL Thomas high performance computing facility (Thomas@UCL).

## 3 Results and discussion

### 3.1 Electrochemical modulation

**FIGURE 2:**
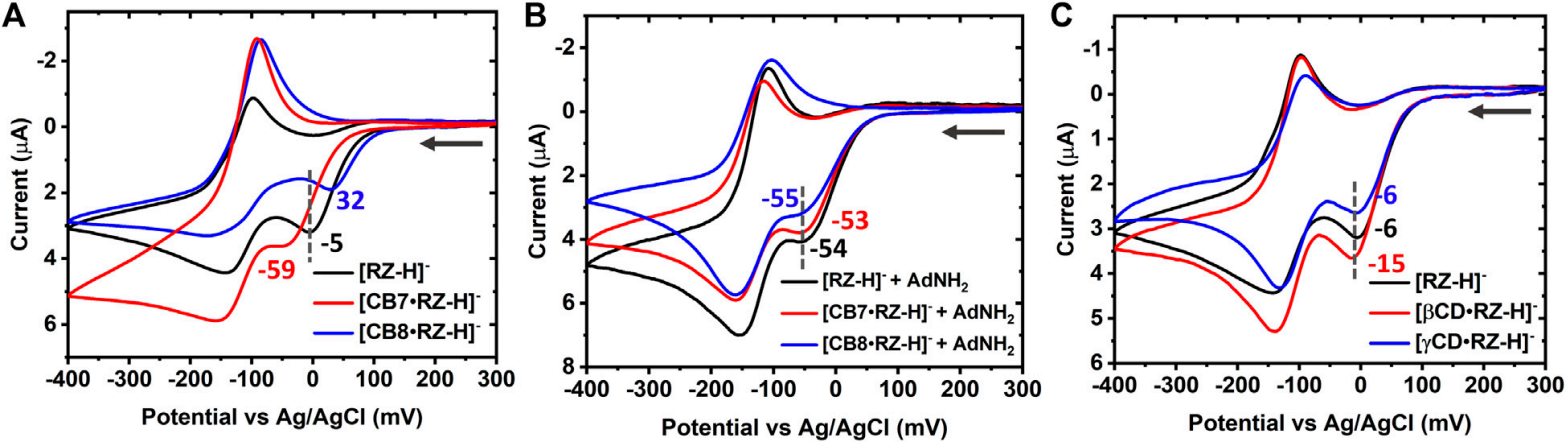
**(A)** Overlaid cyclic voltammograms of 1 mM [RZ-H]^−^ alone (black line), and in the presence of equal molar CB7 (red line) or CB8 (after precipitate removing, blue line) and **(B)** control measurements in the presence of 3 mM of AdNH_2_. **(C)** Overlaid cyclic voltammograms of 1 mM [RZ-H]^−^ alone (black line), and in the presence of equal molar βCD (red line) or γCD (blue line). Electrolyte: B-R buffer (pH 7) solution containing 0.04 M boric acid, 0.04 M phosphoric acid, 0.04 M acetic acid and 0.2 M sodium hydroxide; Potential range: from 300 mV to −400 mV. Scan rate: 10 mV/s. Working electrode: glassy carbon electrode (d = 3 mm).

The effect of supramolecular complexation on the redox properties of [RZ-H]^−^ was investigated using electrochemical techniques, including CV and SWV. It is well-established that RZ undergoes a two-step reduction process: first, through an irreversible two-electron reduction step (*R1*), yielding deprotonated resorufin ([RS-H]^−^), which can subsequently undergo a reversible two-electron reduction step (*R2*) to produce the neutral form of dihydro-resorufin (DHRS), as illustrated in Figure 1A. The cyclic voltammogram of free [RZ-H]^−^ is shown by the black line in Figure 2A, consistent with previous literature reports (Wang et al., 1998). Specifically, the reduction peak potentials of free [RZ-H]^−^ corresponding to *R1* and *R2* are observed at −5 mV and −141 mV vs. Ag/AgCl in CV, respectively.

Upon the introduction of 1 equivalent of CB7 or CB8, a noticeable increase in the peak-to-peak splitting between *R2* and its corresponding anodic peak was observed (Figure 2A), suggesting a reduced diffusivity and a slower electrochemical kinetics within bulky inclusion complexes ([CB7•RZ-H]^−^ and [CB8•RZ-H]^−^) compared to free [RZ-H]^−^ molecules. Furthermore, host-guest complexation with CB7 or CB8 caused distortion in the cathodic waves and a significant increase in the anodic peak current, which can be attributed to slower mass transport kinetics associated with the bulky complexes (Liu et al., 2021). The host-guest complexation/dissociation processes, coupled to the electron-transfer step, have also been reported previously to contribute to the distortions observed in the cathodic wave (Saleh et al., 2008).

Notably, in the presence of 1 equivalent of CB7 or CB8, the irreversible reduction *R1* exhibited opposite shifts with respect to free [RZ-H]^−^: Δ *E*_R1, CB7_ = −54 mV and Δ *E*_R1, CB8_ = +37 mV in CV at pH 7 (see Supplementary Figure S1A for the corresponding SWV data), suggesting that complexation with CB7 impedes the *R1* process of [RZ-H]^−^, whereas complexation with CB8 promotes it. However, no significant reduction peak potential shift was observed in reversible *R2* (Figure 2A; Supplementary Figure S1B), hinting that the preferential binding of CB towards more positively charged species did not play a role in the modulation of [RZ-H]^−^ reactivity. The magnitude of the shift induced by CB in the reduction peak potential *R1* of [RZ-H]^−^ is comparable to the observations in the typical inclusion complex system involving redox-active guests, as reported by Kaifer (Gadde and Kaifer, 2011). For instance, the reduction peak of MV^2+^ undergoes a shift of −30 mV upon complexation with CB7.

Although the reduction potential of RZ is known to be pH-dependent (Wang et al., 1998), the observed modulation of the potential cannot be attributed to a change in the pH of the media in the presence of CB. This is because the pH value of the samples was maintained at 7 throughout the electrochemical measurements using B-R buffer, which also served as the supporting electrolyte. Additionally, this modulation cannot be ascribed to the previously reported p*K*_a_ shift effects of CB (Nau et al., 2011), as no such effect was observed for both [CB7•RZ-H]^−^ and [CB8•RZ-H]^−^ complexes (Supplementary Figure S2). These combined observations indicate that the modulation of the redox activity of [RZ-H]^−^ induced by CB complexation does not occur via a change in the protonation state of [RZ-H]^−^ and thus hinting that it may be caused by alterations in the microenvironment surrounding [RZ-H]^−^ upon complexation with CB.

To investigate the above hypothesis and as a control experiment, 1-adamantylamine (AdNH_2_) was employed as a strong competitive guest to displace [RZ-H]^–^ from the CB cavity. It is known that AdNH_2_ exhibits considerably high binding constants with CB7 and CB8 [*K*_bind_ = 4.23 × 10^12^ M^–1^ for CB7 and *K*_bind_ = 8.19 × 10^8^ M^–1^ for CB8 (Liu et al., 2005)]. Encouragingly, in the presence of 3 equivalents of AdNH_2_, the reduction peak potential corresponding to *R1* in the presence of CB7 or CB8 shifted back to a position almost identical to that of free [RZ-H]^−^ (Figure 2B, see Supplementary Figure S1B for the corresponding SWV data). This finding confirms that the observed modulation effects on redox activity are indeed a result of the formation of host-guest inclusion complexes with CB. Note that we observed a systematic shift towards the negative direction in the reduction peak potential of *R1* in the presence of AdNH_2_. For instance, the *R1* peak potential shifted from −5 mV to −54 mV in the CV (Figures 2A,B). This can be attributed to the formation of a self-assembled monolayer (SAM) of AdNH_2_ on the surface of the glassy carbon electrode, facilitated by hydrophobic interactions between the adamantyl moiety and the mildly hydrophobic glassy carbon surface. The resulting SAM can impede electron transfer from the electrode to resazurin, leading to the systematic shift in reduction potential. Furthermore, the excess cationic Ad-NH_3_^+^ can interact with the anionic [RZ-H]^−^ in the system, which may also contribute to the observed potential shift. Note also that the slight differences in reduction potential between free [RZ-H]^−^ (−54 mV), [CB7•RZ-H]^−^ (−53 mV) and [CB8•RZ-H]^−^ (−55 mV) in Figure 2B can be attributed to the incomplete displacement of [RZ-H]^–^ from the CB7/8 cavities, as host-guest displacement is a dynamic process. Nevertheless, these differences are considered insignificant and within the range of methodological error.

### 3.2 Mechanism analysis

To gain further insights into the modulation mechanism, we conducted measurements using β-cyclodextrin (βCD) and γ-cyclodextrin (γCD) as supramolecular hosts. βCD and γCD are well-known analogues of CB7 and CB8, respectively, with comparable cavity volumes but different geometric structures and interacting functional groups. The formation of inclusion complexes of [RZ-H]^−^ with hosts (CB7, CB8, βCD and γCD) has been studied with ^1^H NMR spectroscopy (Supplementary Figures S2–S6). The down-field shifted RZ signals in the presence of CB7, CB8, and βCD indicates the location of the terminal benzene groups of the guest near the portal region, whereas the middle part (without hydrogen) was included inside the cavity. Interestingly, unlike these three hosts, γCD has caused a significant up-field shift of the guest signals. Fitting of the data suggests a potential 2:1 (guest:host) ternary complexation. The chemical shift of free resazurin drops markedly to 6.23 ppm upon the addition of only 0.5 equiv. of γCD, which is expected to be above 6.30 ppm should it follow a fast-exchange 1:1 binding mechanism (the apparent chemical shift is determined by the content average of the free and complexed guest). The binding affinities of the complexes have been determined via UV-vis titrations and NMR titrations (Table 1; Supplementary Figures S7, S8). It is found that the binding affinities of resazurin with cucurbiturils (6.5 × 10^4^ M^−1^ for [CB7•RZ-H]^−^ and 7.9 × 10^4^ M^−1^ for [CB7•RZ-H]^−^ are generally an order of magnitude larger than their corresponding reference cyclodextrins 2.2×10^3^ M^−1^ for βCD and ca. 10^6^ ∼10^7^ M^−2^ for γCD in the 2:1 complexatin form). Athough the complexaion degree of resazurin is expected to reach over 50% and 90%, respectively, for βCD and γCD in the electrochemical experimental conditions, as calculated from the binidng constants, the shifts observed in *R1*, as shown in Figure 2C and Supplementary Figure S1C, are still relatively small (<10 mV). This indicates that CDs only have minor modulation effects on the electrochemical reduction of [RZ-H]^−^ compared with CBs. This may be attributed to the interacting and geometric differences between the two types of host molecules, including the molecular shape, constrictive binding effect, as well as the different dipole interactions at host portals.

**TABLE 1:** Molecular volume, packing coefficients and binding constants of [CB7•RZ-H]^−^ and [CB8•RZ-H]^−^ with/without one H_2_O molecule included.

	Volume ( Å ^3^)	Packing coefficient (%)		Binding constant (M^–1^)^c^	
		CB7	CB8	CB7	CB8
[RZ-H]^−^	179.8^a^	64 (74)^a^	38 (49)^a^	6.5 × 10^4^	7.9 × 10^4^
	206.29^b^	73 (85)^b^	43 (56)^b^		
[RZ-H]^−^ + H_2_O	196.98^a^	70 (81)^a^	41 (54)^a^	_	_
	225.64^b^	80 (93)^b^	47 (61)^b^		

^a^ Calculated by HyperChem 8.0.10.

^b^ Calculated by Spartan 20 Parallel Suite.

^c^ Obtained by UV-vis, titration; PC, were calculated based on the expanded (inner) cavity volume of CB7 and CB8 reported previously (Nau et al., 2011).

**FIGURE 3:**
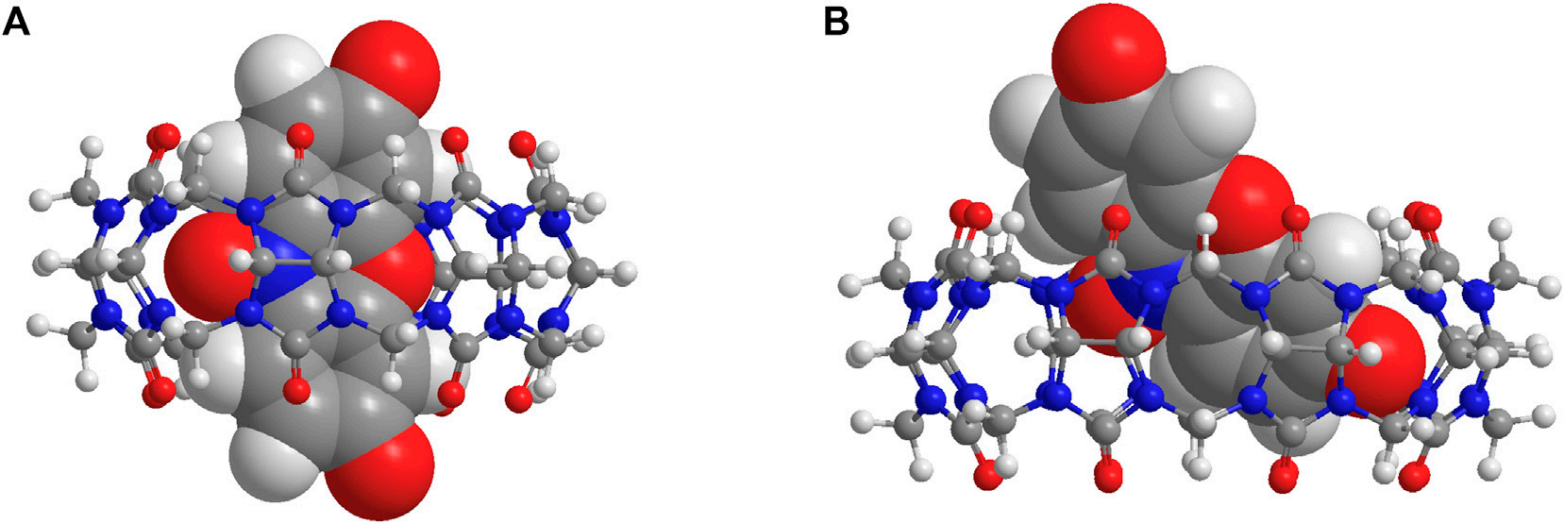
Energy-minimized molecular models of **(A)** [CB7•RZ-H]^–^ and **(B)** [CB8•RZ-H]^–^ at CPCM/wB97XD/6-31G* level of theory.

The irreversible reduction (*R1*) of [RZ-H]^−^ is known to involve cascades of electron transfer and protonation of its -NO group, as illustrated in Figure 1A (Khazalpour and Nematollahi, 2014), and at pH 7, protonation of the -NO group is mediated by water molecules. As reported by the Nematollah et al., the ease of reduction process of [RZ-H]^−^ can be remarkably affected by the ease of -NO group protonation, i.e., the reduction peak potential is shifted to a more positive position as the protonation of -NO is easier to occur at lower pH (Khazalpour and Nematollahi, 2014). Based on this understanding, we propose that the modulation of the electrochemical reactivity of [RZ-H]^−^ is achieved by regulating the accessibility of water molecules to the reaction center (-NO group) for its protonation encapsulated within the CB cavity, as shown in Figure 1B. To support this hypothesis, we studied the binding geometries of the complexes using DFT calculations (Figure 3). The computational results show that the reaction center (the -NO group of [RZ-H]^−^) is deeply immersed in the host cavity for both CB7 and CB8. Therefore, the accessibility of water molecules to the confined space where the -NO group is located becomes a key factor for the reduction of [RZ-H]^–^, as shown in the energy-minimized models in Supplementary Figure S9.

To evaluate the tightness of the binding of RZ in both host molecules, we calculated the van der Waals volume of [RZ-H]^−^ using HyperChem software and Spartan software, and determined its packing coefficient (PC) within CB7 and CB8 (Table 1). The PC of [RZ-H]^−^ in CB7, concerning both the expanded and inner cavity volume, appears to be larger than the optimum value of 55% as defined in Rebek’s rule (Mecozzi and Rebek, 1998). However, considering that [RZ-H]^−^ resides only partially in the cavity rather than being fully encapsulated, as shown in Figure 3, the calculated PC values, which use the full volume of the guest molecules, are overestimates. In the case of CB8, the PC generally falls around 55%, even with a partial complexation pattern, indicating that the remaining void space inside the host cavity can remain certain moving freedom (*e.g.*, vibration, rotation, even translation) for both host and guest molecules and even allows the entry of other small molecules, such as a water molecule. This is in contrast to CB7, where tight binding may restrict the accessibility of water molecules to approach the -NO group at the cavity center (Figure 3A).

As a consequence, the conversion from [RZ-H]^−^ to [RS-H]^−^ requires partial displacement of [RZ-H]^−^ from the CB7 cavity for both [CB7•RZ-H]^−^ and its one-electron reduced intermediate [CB7•RZ-H + e]^2−^. This can be observed in their optimized molecular models in Supplementary Figures S9A–S10B, respectively. The partial dissociation is expected to weaken the host-guest interactions, thus destabilizing the reaction intermediate during the proton transfer step. On the other hand, in the case of CB8, the larger cavity size ensures no significant change in the binding geometry before and after the introduction of a water molecule around the -NO group, both for [CB8•RZ-H]^−^ (Supplementary Figure S9B) and [CB8•RZ-H + e]^2−^ (Supplementary Figure S10D). This suggests the presence of pre-organization effects in [CB8•RZ-H]^−^ and [CB8•RZ-H + e]^2−^, which effectively accommodate and stabilize the incoming water molecule during the proton transfer step. This pre-organization effect may also contribute to the decrease in entropic penalty upon encapsulation of a water molecule.

To gain semi-quantitative insights into the hypothesis, we calculated energy values of the optimized structures of the one-electron reduced intermediates (free [RZ-H + e]^2−^, [CB7•RZ-H + e]^2−^ and [CB8•RZ-H + e]^2−^) interacting with a water molecule located at two different sites: the phenoxide group or the -NO group (Supplementary Figure S10; Supplementary Table S1). For free [RZ-H + e]^2−^, the system experiences a slight destabilization of +2.7 kcal mol^–1^ when the water molecule moves from the phenoxide group to the–NO group. Importantly, this energy difference increases to +6.8 kcal mol^–1^ in the case of [CB7•RZ-H + e]^2−^, indicating a decreased energetic favorability for the water molecule to approach the reaction center. Conversely, the energy difference reverses to −4.2 kcal mol^–1^ in the case of [CB8•RZ-H + e]^2−^, an energetically favorable approach of the water molecule, which supports our hypothesis. We acknowledge that the current DFT modelling results do not account for the entropic terms which can be important in these systems and can be a subject for further computational investigation.

## 4 Conclusion

In summary, we have successfully demonstrated the deactivation and activation of the electrochemical reactivity of resazurin through host-guest complexation with rigid molecular hosts CB7 and CB8, respectively. This modulation effect is attributed to the tightness of the binding, determined by the combined effects of the packing coefficient and constrictive binding, which control the accessibility of water molecules to the encapsulated reaction center during the proton transfer step. Notably, flexible host molecules βCD and γCD do not exhibit reactivity modulation, highlighting the unique role of CB’s well-defined rigid cavity and their structural features. These findings offer opportunities for fine-tuning the redox behavior of resazurin to accommodate a wider range of target molecules in biosensing applications. Furthermore, our generic approach can be extended to other redox-active processes involving proton transfer and is expected to inspire further understanding and design of nano-confined chemical systems.

## Data Availability

The original contributions presented in the study are included in the article/SupplementaryMaterial, further inquiries can be directed to the corresponding authors.

## References

[B1] Barrow S. J. Kasera S. Rowland M. J. Del Barrio J. Scherman O. A. (2015). Cucurbituril-based molecular recognition. Chem. Rev. 115, 12320–12406. 10.1021/acs.chemrev.5b00341 26566008

[B2] Boulas P. L. Gómez‐Kaifer M. Echegoyen L. (1998). Electrochemistry of supramolecular systems. Angew. Chem. Int. Ed. 37, 216–247. 10.1002/(sici)1521-3773(19980216)37:3<216::aid-anie216>3.0.co;2-p 29711273

[B3] Cai K. Zhang L. Astumian R. D. Stoddart J. F. (2021). Radical-pairing-induced molecular assembly and motion. Nat. Rev. Chem. 5, 447–465. 10.1038/s41570-021-00283-4 37118435

[B4] Chio W.-I. K. Liu J. Jones T. Perumal J. Dinish U. S. Parkin I. P. (2021). SERS multiplexing of methylxanthine drug isomers via host–guest size matching and machine learning. J. Mater. Chem. C 9, 12624–12632. 10.1039/D1TC02004H

[B5] Chio W.-I. K. Moorthy S. Perumal J. U. S D. Parkin I. P. Olivo M. (2020). Dual-triggered nanoaggregates of cucurbit[7]uril and gold nanoparticles for multi-spectroscopic quantification of creatinine in urinalysis. J. Mater. Chem. C 8, 7051–7058. 10.1039/D0TC00931H

[B6] Chio W.-I. K. Xie H. Zhang Y. Lan Y. Lee T.-C. (2022). SERS biosensors based on cucurbituril-mediated nanoaggregates for wastewater-based epidemiology. Trends Anal. Chem. 146, 116485. 10.1016/j.trac.2021.116485

[B7] Chio W. K. Peveler W. J. Assaf K. I. Moorthy S. Nau W. M. Parkin I. P. (2019). Selective detection of nitroexplosives using molecular recognition within self-assembled plasmonic nanojunctions. J. Phys. Chem. C 123, 15769–15776. 10.1021/acs.jpcc.9b02363 PMC6614880 31303905

[B8] Davison G. Jones T. Liu J. Kim J. Yin Y. Kim D. (2023). Computer-aided design and Analysis of spectrally aligned hybrid plasmonic nanojunctions for SERS detection of nucleobases. Adv. Mater. Technol. 8, 2201400. 10.1002/admt.202201400

[B9] Emter R. Natsch A. (2015). A fast Resazurin-based live viability assay is equivalent to the MTT-test in the KeratinoSens assay. Toxicol. Vitro. 29, 688–693. 10.1016/j.tiv.2015.02.003 25687527

[B10] Gadde S. Batchelor E. K. Kaifer A. E. (2009). Controlling the formation of cyanine dye H- and J-aggregates with cucurbituril hosts in the presence of anionic polyelectrolytes. Chemistry 15, 6025–6031. 10.1002/chem.200802546 19402091

[B11] Gadde S. Kaifer A. (2011). Cucurbituril complexes of redox active guests. Curr. Org. Chem. 15, 27–38. 10.2174/138527211793797800

[B12] Ibáñez D. Izquierdo-Bote D. Pérez-Junquera A. González-García M. B. Hernández-Santos D. Fanjul-Bolado P. (2020). Raman and fluorescence spectroelectrochemical monitoring of resazurin-resorufin fluorogenic system. Dyes Pigm 172, 107848. 10.1016/j.dyepig.2019.107848

[B13] Isaacs L. (2009). Cucurbit [n] urils: from mechanism to structure and function. Chem. Comm. 6, 619–629. 10.1039/b814897j 19322405

[B14] Jeon W. S. Kim H.-J. Lee C. Kim K. (2002). Control of the stoichiometry in host–guest complexation by redox chemistry of guests: inclusion of methylviologen in cucurbit [8] uril. Chem. Comm. 17, 1828–1829. 10.1039/b202082c 12271629

[B15] Khazalpour S. Nematollahi D. (2014). Electrochemical study of Alamar Blue (resazurin) in aqueous solutions and room-temperature ionic liquid 1-butyl-3-methylimidazolium tetrafluoroborate at a glassy carbon electrode. RSC Adv. 4, 8431–8438. 10.1039/C3RA45800H

[B16] Kim H.-J. Jeon W. S. Ko Y. H. Kim K. (2002). Inclusion of methylviologen in cucurbit [7] uril. Proc. Natl. Acad. Sci. U.S.A. 99, 5007–5011. 10.1073/pnas.062656699 11917115 PMC122712

[B17] Lee T. C. Kalenius E. Lazar A. I. Assaf K. I. Kuhnert N. Grün C. H. (2013). Chemistry inside molecular containers in the gas phase. Nat. Chem. 5, 376–382. 10.1038/nchem.1618 23609087

[B18] Lin R.-L. Liu J.-X. Chen K. Redshaw C. (2020). Supramolecular chemistry of substituted cucurbit[n]urils. Inorg. Chem. Front. 7, 3217–3246. 10.1039/D0QI00529K

[B19] Ling Y. Mague J. T. Kaifer A. E. (2007). Inclusion complexation of diquat and paraquat by the hosts cucurbit [7] uril and cucurbit [8] uril. Chem. Eur. J. 13, 7908–7914. 10.1002/chem.200700402 17685378

[B20] Liu J. Lambert H. Zhang Y.-W. Lee T.-C. (2021). Rapid estimation of binding constants for cucurbit [8] uril ternary complexes using electrochemistry. Anal. Chem. 93, 4223–4230. 10.1021/acs.analchem.0c04887 33595296 PMC8023530

[B21] Liu S. Ruspic C. Mukhopadhyay P. Chakrabarti S. Zavalij P. Y. Isaacs L. (2005). The cucurbit [n] uril family: prime components for self-sorting systems. J. Am. Chem. Soc. 127, 15959–15967. 10.1021/ja055013x 16277540

[B22] Luo H. Chen L.-X. Ge Q.-M. Liu M. Tao Z. Zhou Y.-H. (2019). Applications of macrocyclic compounds for electrochemical sensors to improve selectivity and sensitivity. J. Incl. Phenom. Macrocycl. Chem. 95, 171–198. 10.1007/s10847-019-00934-6

[B23] Mecozzi S. Rebek J. (1998). The 55 % solution: a formula for molecular recognition in the liquid state. Chem. Eur. J. 4, 1016–1022. 10.1002/(SICI)1521-3765(19980615)4:6<1016::AID-CHEM1016>3.0.CO;2-B

[B24] Mishra P. Singh D. Mishra K. P. Kaur G. Dhull N. Tomar M. (2019). Rapid antibiotic susceptibility testing by resazurin using thin film platinum as a bio-electrode. J. Microbiol. Methods. 162, 69–76. 10.1016/j.mimet.2019.05.009 31103460

[B25] Moorthy S. Castillo Bonillo A. Lambert H. Kalenius E. Lee T.-C. (2022). Modulating the reaction pathway of phenyl diazonium ions using host–guest complexation with cucurbit[7]uril. Chem. Comm. 58, 3617–3620. 10.1039/D1CC06982A 35199806

[B26] Moorthy S. Lambert H. Mohan N. Schwarzlose T. Nau W. M. Kalenius E. (2023). Noncovalent modulation of chemoselectivity in the gas phase leads to a switchover in reaction type from heterolytic to homolytic to electrocyclic cleavage. Angew. Chem. Int. Ed. 62, e202303491. 10.1002/anie.202303491 37161709

[B27] Nau W. M. Florea M. Assaf K. I. (2011). Deep inside cucurbiturils: physical properties and volumes of their inner cavity determine the hydrophobic driving force for host–guest complexation. Isr. J. Chem. 51, 559–577. 10.1002/ijch.201100044

[B28] O'Brien J. Wilson I. Orton T. Pognan F. (2000). Investigation of the Alamar Blue (resazurin) fluorescent dye for the assessment of mammalian cell cytotoxicity. Eur. J. Biochem. 267, 5421–5426. 10.1046/j.1432-1327.2000.01606.x 10951200

[B29] Ong W. Gómez-Kaifer M. Kaifer A. E. (2002). Cucurbit [7] uril: a very effective host for viologens and their cation radicals. Org. Lett. 4, 1791–1794. 10.1021/ol025869w 12000300

[B30] Präbst K. Engelhardt H. Ringgeler S. Hübner H. (2017). Basic colorimetric proliferation assays: MTT, WST, and resazurin. Methods Mol. Biol. 1601, 1–17. 10.1007/978-1-4939-6960-9_1 28470513

[B31] Saleh N. Koner A. L. Nau W. M. (2008). Activation and stabilization of drugs by supramolecular p*K* _a_ shifts: drug‐delivery applications tailored for cucurbiturils. Angew. Chem. Int. Ed. 120, 5478–5481. 10.1002/ange.200801054 18548429

[B32] Uhlenheuer D. A. Petkau K. Brunsveld L. (2010). Combining supramolecular chemistry with biology. Chem. Soc. Rev. 39, 2817–2826. 10.1039/B820283B 20461247

[B33] Walker S. Oun R. McInnes F. J. Wheate N. J. (2011). The potential of cucurbit[n]urils in drug delivery. Isr. J. Chem. 51, 616–624. 10.1002/ijch.201100033

[B34] Wang Y. Mendoza S. Kaifer A. E. (1998). Electrochemical reduction of cobaltocenium in the presence of β-cyclodextrin. Inorg. Chem. 37, 317–320. 10.1021/ic970702y

