# Supplementary material for: Modulation of redox reactivity of resazurin through host-guest complexation with Cucurbit[n]uril (n = 7, 8)
Source: Front Chem. 2023 Dec 14;11:1295715. doi: 10.3389/fchem.2023.1295715 (PMC10755866; doi:10.3389/fchem.2023.1295715)
Supplement: Supplementary file 1 [file DataSheet1.pdf]

Supplementary Material

TABLE OF CONTENTS

Figure S1. Overlaid square wave voltammograms of 'free $[RZ\text{-}H]^-$, $[CB7•RZ\text{-}H]^-$ and $[CB8•RZ\text{-}H]^-$' and 'free $[RZ\text{-}H]^-$, $[\beta CD•RZ\text{-}H]^-$ and $[\gamma CD•RZ\text{-}H]^-$'..2
Figure S2. UV-vis absorption spectra of RZ, CB7•RZ and CB8•RZ at different pH…............3
Figure S3. ^{1}H NMR spectra of $[RZ\text{-}H]^-$ and $[\beta CD•RZ\text{-}H]^-$……...4
Figure S4. ^{1}H NMR spectra of $[RZ\text{-}H]^-$ and $[\gamma CD•RZ\text{-}H]^-$……...4
Figure S5. ^{1}H NMR spectra of $[RZ\text{-}H]^-$ and $[CB7•RZ\text{-}H]^-$..…...5
Figure S6. ^{1}H NMR spectra of $[RZ\text{-}H]^-$ and $[CB8•RZ\text{-}H]^-$…..5
Figure S7. UV-vis spectra of 4 μM $[RZ\text{-}H]^-$ in water upon the gradual addition of host molecules CB7/CB8/βCD/γCD...6
Figure S8. ^{1}H NMR spectra of 1 mM $[RZ\text{-}H]^-$ in the presence of different equivalents of βCD and γCD...7
Figure S9. Energy-minimized molecular models of $[CB7•RZ\text{-}H]^-$ and $[CB8•RZ\text{-}H]^-$ with/without H_2O molecule near reaction center -NO………..8
Figure S10. Energy-minimized molecular models $[CB7•RZ\text{-}H\text{+}e]^{2-}$ and $[CB8•RZ\text{-}H\text{+}e]^{2-}$ with one H_2O molecule at different sites ………..8
Table S1. Optimized energies of free $[RZ\text{-}H\text{+}e]^{2-}$, $[CB7•RZ\text{-}H\text{+}e]^{2-}$ and $[CB8•RZ\text{-}H\text{+}e]^{2-}$ interacting with one H_2O molecule..………...9

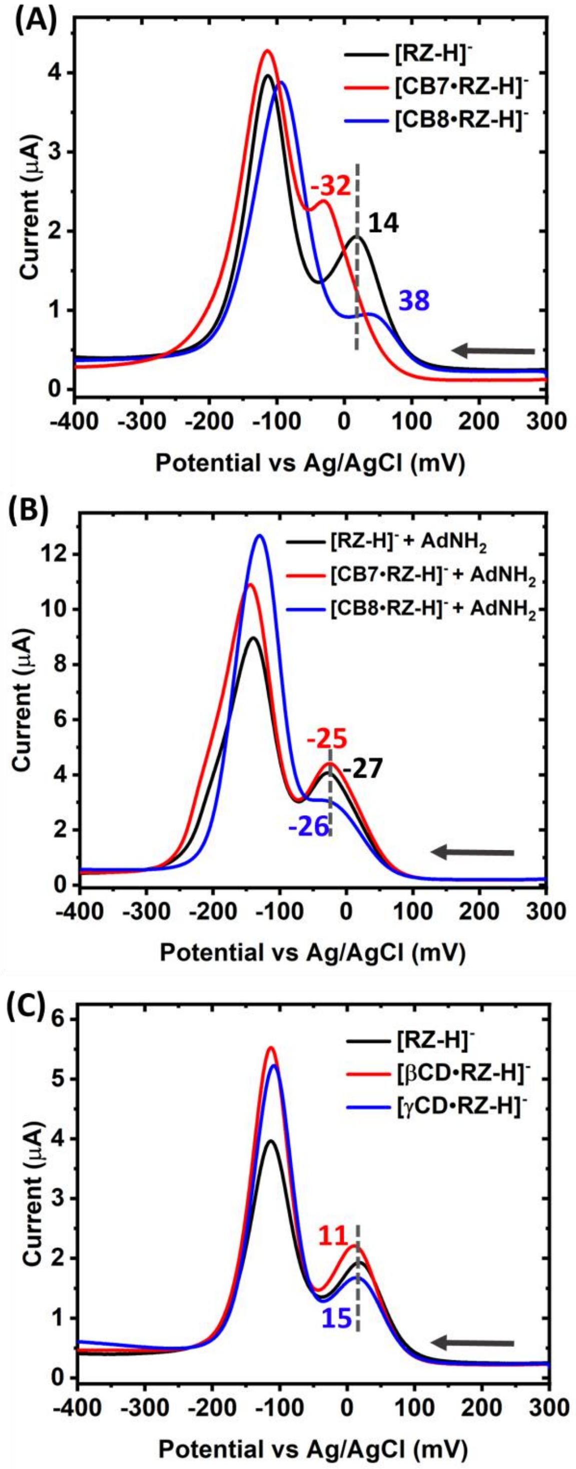

Supplementary Figure S1. (A) Overlaid square wave voltammograms of 1 mM $[RZ\text{-}H]^-$ alone (black line) and in the presence of equal molar of CB7 (red line) and CB8 (blue line) and **(B)** control measurements in the presence of 3 mM of $AdNH_2$. **(C)** Overlaid square wave voltammograms of 1 mM $[RZ\text{-}H]^-$ alone (black line) and in the presence of equal molar βCD (red line) and γCD (blue line).

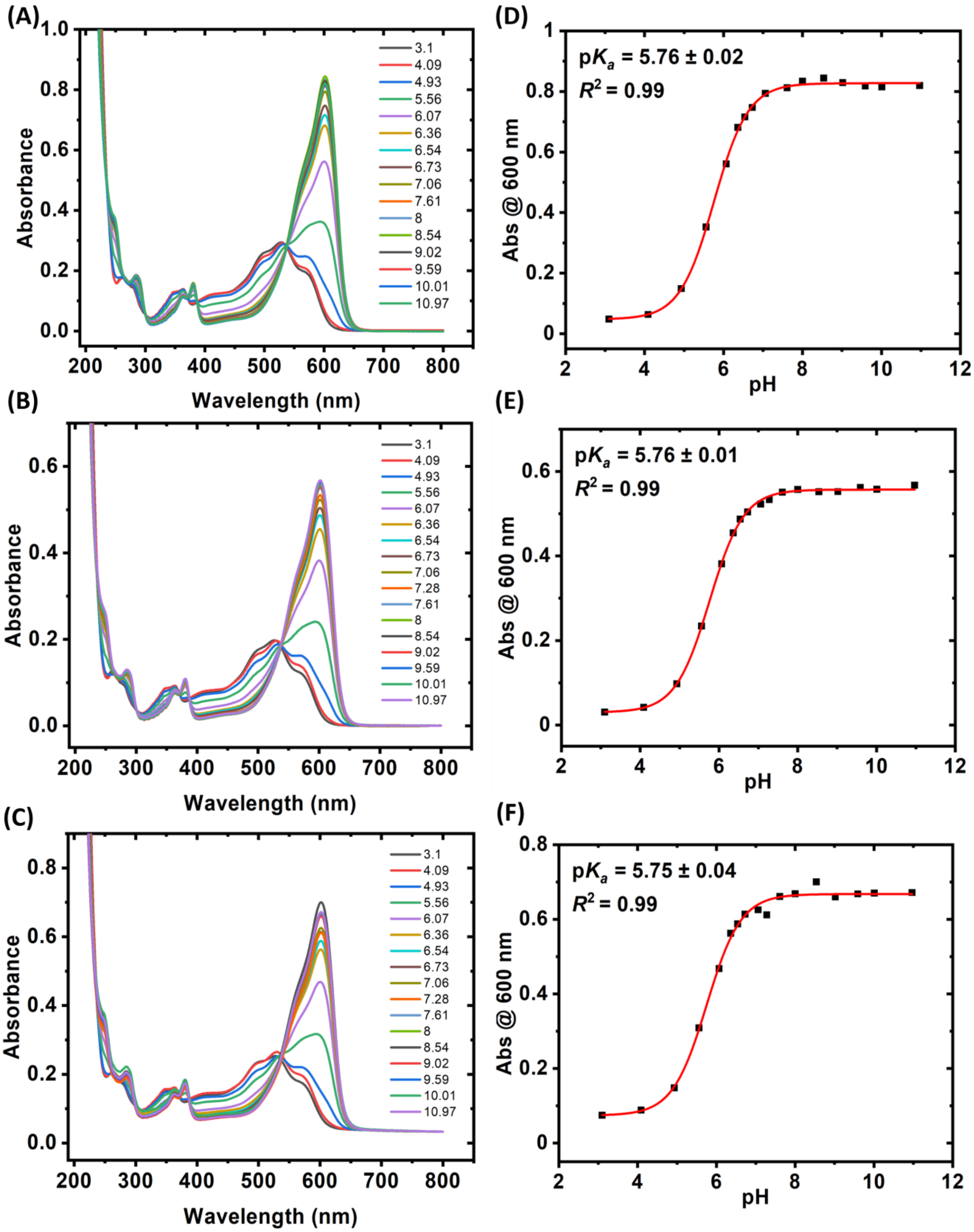

Supplementary Figure S2. UV-vis absorption spectra of 20 μM **(A)** RZ **(B)** CB7•RZ and **(C)** CB8•RZ at different pH ranging from 3.1 to 10.97 in B-R buffer. pK_a titration fitting results of **(D)** RZ, **(E)** CB7•RZ and **(F)** CB8•RZ obtained by using the absorption peak at 600 nm.

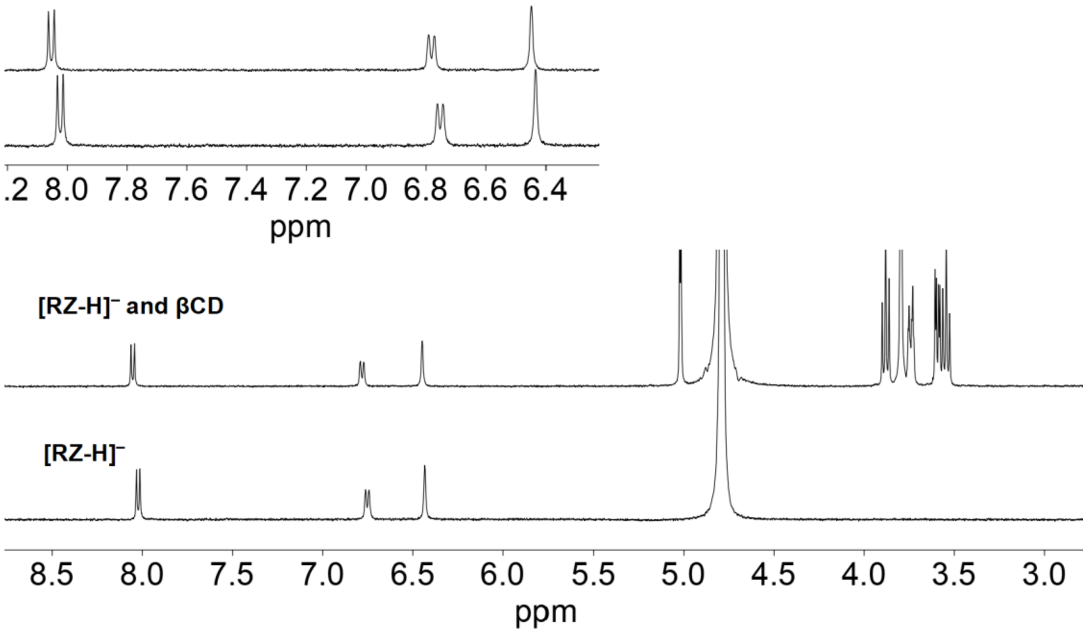

Supplementary Figure S3. ^{1}H NMR spectra of 1mM $[RZ\text{-}H]^{-}$ in the absence (bottom) and presence (upper) of 1 mM βCD in D_2O. The chemical shift range from 6.3 ppm to 8.1 ppm has been zoomed in for better visualization.

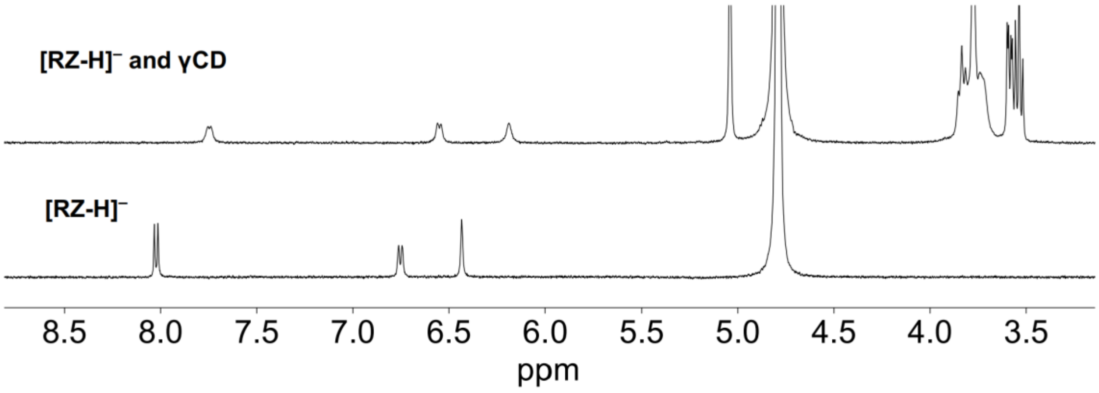

Supplementary Figure S4. ^{1}H NMR spectra of 1mM $[RZ\text{-}H]^{-}$ in the absence (bottom) and presence (upper) of 1 mM γCD in D_2O.

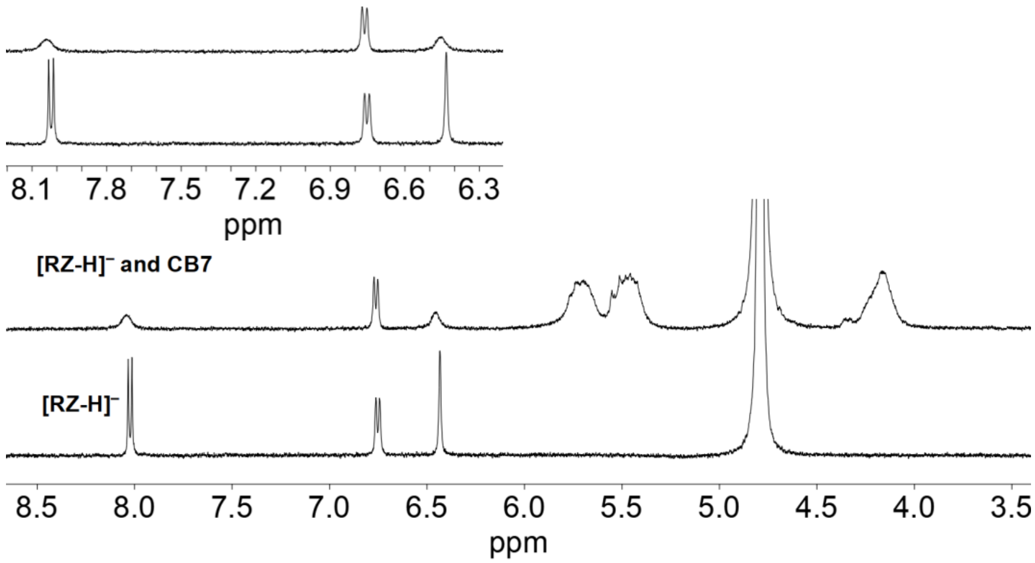

Supplementary Figure S5. ^{1}H NMR spectra of 1mM [RZ-H]⁻ in the absence (bottom) and presence (upper) of 1 mM CB7 in D_2O. The chemical shift range from 6.3 ppm to 8.1 ppm has been zoomed in for better visualization.

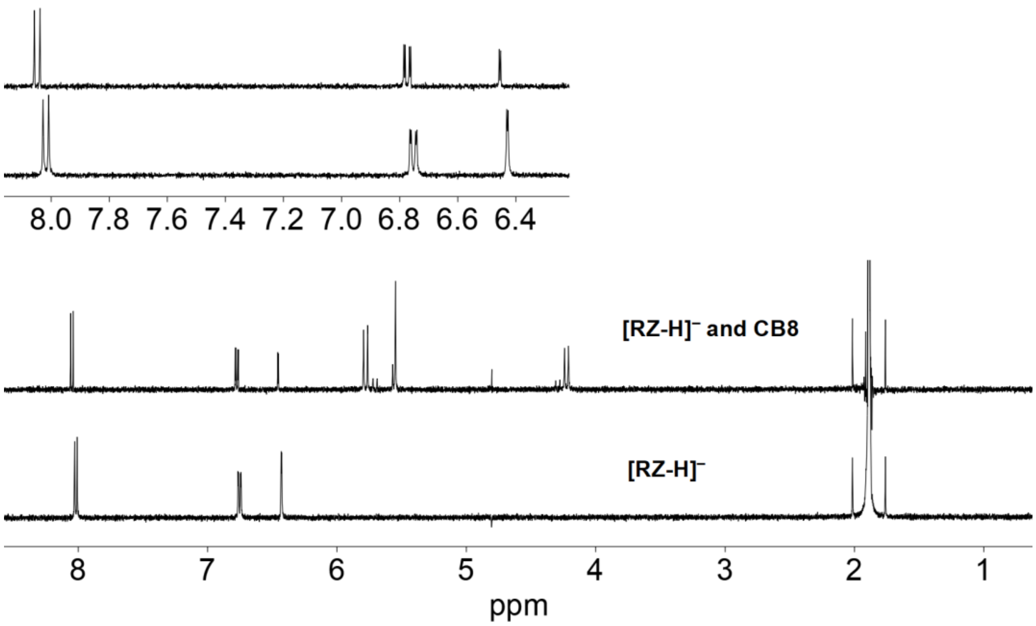

Supplementary Figure S6. ^{1}H NMR spectra of 1mM [RZ-H]⁻ in the absence (bottom) and presence (upper) of 1 mM CB8. Solvent: 40 mM mixture of sodium borate, sodium phosphate and sodium acetate in D_2O. Water suppression has been applied. Please note that precipitate was formed when mixing guest and host, and it was filtered before measurement. The chemical shift range from 6.3 ppm to 8.1 ppm has been zoomed in for better visualization.

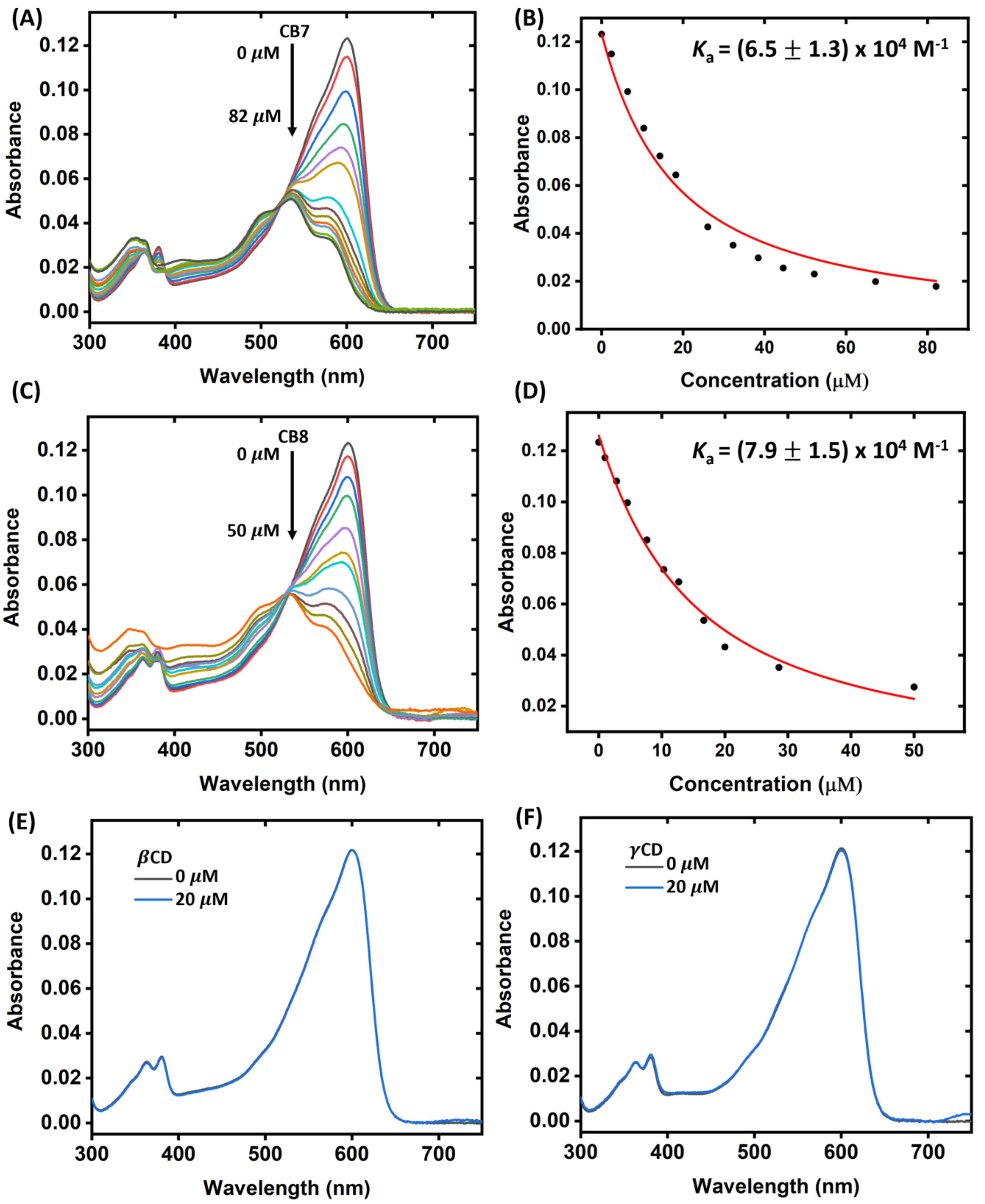

Supplementary Figure S7. UV-vis spectra of 4 μM $[RZ\text{-}H]^-$ in water upon the gradual addition of host molecules: **(A)** CB7, **(C)** CB8, **(E)** βCD, and **(F)** γCD, along with the corresponding titration curves for **(B)** CB7 and **(D)** CB8 using the absorbance at the wavelength of 600 nm.

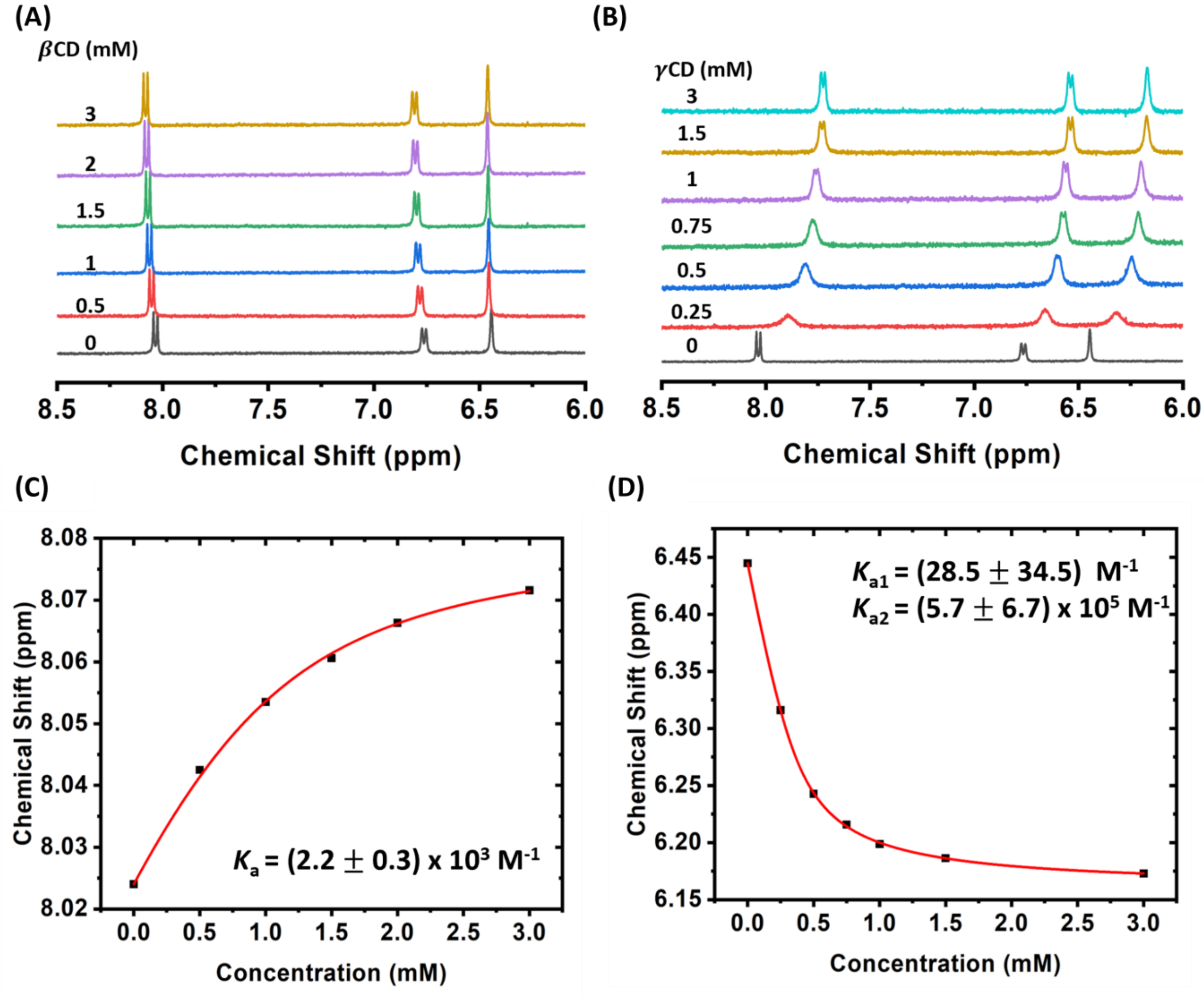

Supplementary Figure S8. ^{1}H NMR spectra of 1 mM [RZ-H]$^{-}$ in the presence of different equivalents of **(A)** βCD and **(B)** γCD, along with the corresponding titration curves for **(C)** βCD and **(D)** γCD. Note that the binding ratio between [RZ-H]$^{-}$ and γCD is likely to be 1:2 according to the titration data. The binding constant of the overall 1:2 complex is estimated to be 10^{6} ~ 10^{7} M^{-2}.

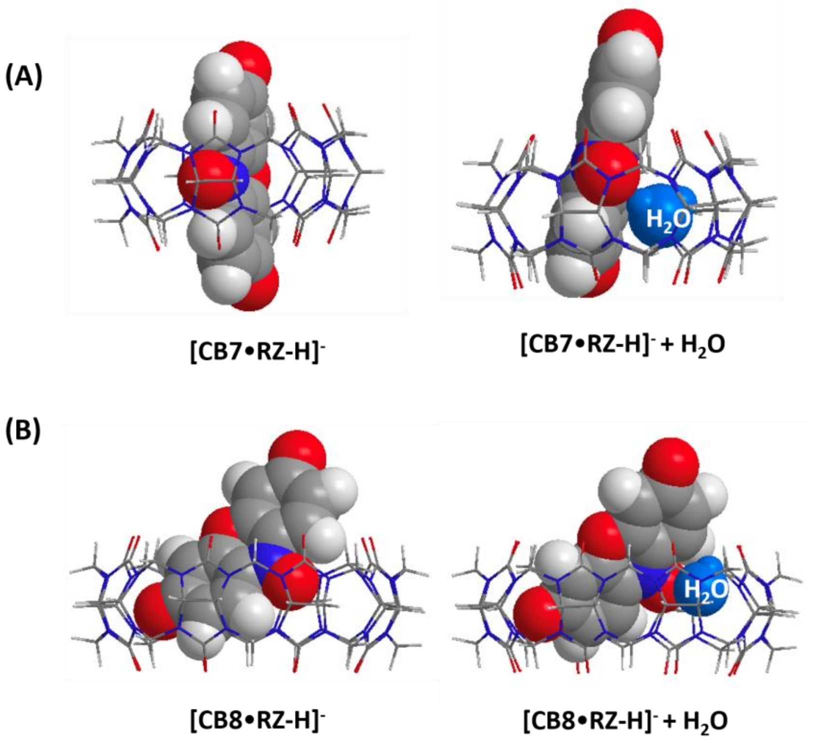

Supplementary Figure S9. Energy-minimized molecular models of **(A)** [CB7•RZ-H]$^{–}$ and **(B)** [CB8•RZ-H]$^{–}$ with/without H_2O molecule near reaction center -NO at CPCM/wB97XD/6-31G* level of theory.

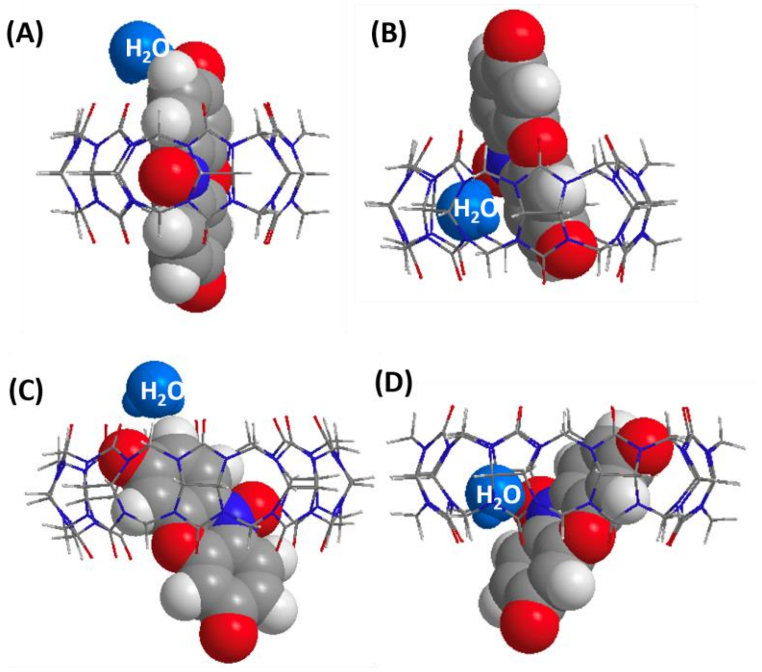

Supplementary Figure S10. Energy-minimized molecular models of [CB7•RZ-H+e]$^{2–}$ with H_2O molecule **(A)** near -O$^{–}$, **(B)** near reaction center -NO. Energy-minimized molecular models of [CB8•RZ-H+e]$^{2–}$ with H_2O molecule **(C)** near -O$^{–}$, **(D)** near reaction center -NO.

Supplementary Table S1. Optimized energies of free $[RZ\text{-}H+e]^{2-}$, $[CB7\bullet RZ\text{-}H+e]^{2-}$ and $[CB8\bullet RZ\text{-}H+e]^{2-}$ interacting with one H_2O molecule computed at CPCM/wB97XD/6-31G* level of theory.

	Position of H_2O	Optimized Energy (kcal/mol)	ΔE (kcal/mol)
$[RZ\text{-}H+e]^{2-}$ + H_2O	Near -O^-	E_O = –560269.7699	+2.7
	Near -NO	E_N = –560267.0987	
$[CB7\bullet RZ\text{-}H+e]^{2-}$ + H_2O	Near -O^-	E_O = –3202902.01	+6.8
	Near -NO	E_N = –3202895.236	
$[CB8\bullet RZ\text{-}H+e]^{2-}$+ H_2O	Near -O^-	E_O = –3580409.198	–4.2
	Near -NO	E_N = –3580413.347	
